# Notch Signaling Is Associated with LED Light-Regulated Papilla Regeneration in the Sea Cucumber (*Apostichopus japonicus*)

**DOI:** 10.3390/ijms27094105

**Published:** 2026-05-04

**Authors:** Dongyao Cui, Yi Wang, Jianpin Xia, Yu Dou, Jingxian Sun, Yaqing Chang

**Affiliations:** 1 College of Biological Science and Technology, Shenyang Agricultural University, Shenyang 110866, China; dloucdy0915@163.com; 2 Key Laboratory of Mariculture & Stock Enhancement in North China’s Sea, Ministry of Agriculture and Rural Affairs, Dalian Ocean University, Dalian 116023, China; 3 College of Ocean and Earth Sciences, Xiamen University, Xiamen 361102, China

**Keywords:** sea cucumber, regeneration, Notch signaling, LED light, cell proliferation

## Abstract

The tissue regeneration of sea cucumber (*Apostichopus japonicus*) involves precise intercellular signal pathway transduction and gene expression regulation. This study investigated the function of the Notch signaling pathway in *A. japonicus* papilla regeneration and its modulation by LED light of varying intensities. We detected the expression patterns of Notch signaling pathway-related genes and their downstream cell proliferation-related genes during papilla regeneration, and further verified the pathway function via gene silencing, combined with histological analyses to explore LED-mediated effects. Gene expression assays revealed that *AjNotch*, *AjSu(H)*, *AjHes1*, *AjCyclinA*, *AjCyclinD* and *AjCDK8* were significantly upregulated at 28 days post papilla excision (*p* < 0.05). LED light treatment accelerated papilla regeneration in a light intensity-dependent manner, with the most pronounced promotion at 2000 lx (*p* < 0.05). Moreover, LED light treatment was associated with altered expression of Notch signaling pathway genes and their downstream proliferation-related genes in a light intensity-dependent manner. Gene silencing of *AjNotch* significantly downregulated its downstream target genes (*p* < 0.05), attenuated the regenerative promotion of LED light, and reduced cell proliferation rate (*p* < 0.05). These findings suggest that the Notch signaling pathway is pivotal for *A. japonicus* papilla regeneration, and LED light modulates papilla regeneration with concurrent changes in the expression of Notch pathway-related genes. This study provides novel insights into the function of the Notch signaling pathway in echinoderm regenerative development.

## 1. Introduction

The edible sea cucumber *Apostichopus japonicus* (Holothuroidea, Echinodermata) is one of the most economically important farmed echinoderms in Asian countries [1]. Driven by the growing interest in its nutritional and medicinal value, the aquaculture scale of this species has expanded significantly [2,3,4]. Notably, in China, the total output of cultured *A. japonicus* exceeded 320,000 tons in 2024, further highlighting its prominent status in the aquaculture industry [5]. Meanwhile, owing to its strong regenerative capacity, *A. japonicus* has become an ideal biological model for studying metazoan regeneration mechanisms, developmental regulation and functional genes, which is of great theoretical significance for revealing the nature of regeneration and inspiring research in regenerative medicine [6,7]. Numerous studies have demonstrated the remarkable regenerative capacity of the sea cucumber *A. japonicus* [8,9]. Upon body wall injury, internal organ evisceration, or organ loss, this species can efficiently initiate repair processes involving cell dedifferentiation, bud formation, and tissue remodeling [10,11,12]. As a vital organ responsible for osmotic pressure regulation and defense, the body wall plays a crucial role in the normal physiological function of *A. japonicus* [13]. Additionally, the rapid regeneration of internal organs serves as a fundamental survival strategy for *A. japonicus* to cope with environmental stressors [14].

The papilla, an accessory organ on the body wall of *A. japonicus*, is a terminal extension of its water vascular system. This system is a unique hydraulic tubular network of echinoderms that regulates appendage movement via coelomic fluid pressure and mediates key physiological processes such as locomotion, attachment, respiration and immunity, through which the papilla fulfills the sea cucumber’s respiratory, defensive and sensory functions [3,15,16]. Beyond these critical physiological roles, the quantity and morphological uniformity of papillae on the body wall also serve as important phenotypic traits for evaluating variety superiority, with higher numbers and plump, regular papillae generally representing healthier individuals and higher-quality germplasm in farmed sea cucumbers [17,18]. During the cultivation and transportation of *A. japonicus*, poor water quality and physical damage often lead to abnormal development or damage of papillae [19,20]. Failure to intervene promptly can result in papilla ulceration caused by bacterial infections, which may trigger the widespread occurrence of skin ulceration syndrome (SUS) [21,22]. This directly affects the product quality and breeding benefits of *A. japonicus*. However, unlike the regeneration of the body wall and internal organs, the regulatory mechanisms underlying papilla regeneration in *A. japonicus* remain poorly explored.

Echinoderm regeneration is governed by key biological processes including cell fate determination, with the Notch signaling pathway as a core regulator via coordination with other genes [23,24]. By mediating intercellular communication, it modulates stem cell fate through a conserved cascade: transmembrane Notch receptors bind adjacent Delta/Serrate/Jagged ligands, triggering cleavage to release the active Notch intracellular domain (NICD) [25]. NICD translocates to the nucleus, binds to Su(H)/RBP-Jκ/CBF1, and forms an activation complex to induce the expression of downstream bHLH repressors, among which Hes/Hey proteins are core members of the repressive bHLH factor subfamily [26,27]. Subsequently, Hes/Hey proteins regulate cell proliferation, differentiation, and undifferentiated states by antagonizing activating bHLH factors, which enables the Notch pathway to fine-tune cell fate during echinoderm regeneration [28,29]. Evidence from the mesoderm and endoderm of sea urchins and sea stars confirms the pivotal role of the Notch signaling pathway in regulating regeneration through lateral inhibition or induced mode switching [24,30]. Our previous transcriptomic analysis in *A. japonicus* identified three genes, *AjNotch*, *AjSu(H)*, and *AjHes1*, which were significantly upregulated during tube foot regeneration, indicating their potential roles in this process. Current research on papilla regeneration has mainly focused on morphology and transcriptomics, and several pathways including Notch, fibroblast growth factor (FGF), and extracellular matrix–related pathways have been implicated, yet the complete regulatory network remains unclear [31]. While Notch signaling is well studied in echinoderm arm regeneration, its expression pattern, mechanism, and functions in cell proliferation and differentiation during sea cucumber papilla regeneration are still poorly understood [32]. Further experimental investigation of Notch signaling and its downstream targets in tube foot regeneration is therefore of great importance.

Seawater environmental factors, including temperature, salinity, and light, are critical regulators of growth, feeding, and tissue repair in aquatic animal aquaculture [33,34,35,36]. As an eco-friendly and scalable physical method, LED light promotes wound healing in turbot and other aquatic animals by modulating inflammatory factors and matrix metalloproteinases [36,37]. In adult *A. japonicus*, LED light regulates physiology via tissue-expressed opsins, thereby affecting feeding and growth [38]. In echinoderms, light signals sensed by opsins may couple with G proteins and transduce downstream through the cAMP-PKA-CREB axis, activating ERK1/2 and Notch signaling [38]. Notch signaling is evolutionarily conserved and governs cell fate decisions in both vertebrates and echinoderms. In vertebrates, it mainly mediates development and tissue homeostasis, whereas echinoderms strongly rely on Notch to drive appendage regeneration, blastema formation, and tissue reconstruction [39,40]. Notably, light can induce oscillatory Notch activity and regulate the neurogenic potential of neural progenitors, suggesting that light may modulate regeneration through Notch crosstalk [41]. Echinoderms possess a unique light-sensing system, and *A. japonicus* shows clear negative phototaxis in locomotion and feeding that is highly sensitive to light intensity [38]. However, it remains unclear whether LED light regulates papilla regeneration in *A. japonicus* by targeting components of the Notch signaling pathway.

In this study, we analyzed the expression patterns of Notch signaling pathway genes (*AjNotch*, *AjSu(H)*, and *AjHes1*) and cell cycle-related genes (*AjCyclinA*, *AjCyclinD*, and *AjCDK8*) during papilla regeneration and under different LED light intensities in *A. japonicus* [42,43]. Using siRNA-mediated *AjNotch* silencing combined with histological staining and EdU cell proliferation assays, we investigated the role of the Notch pathway in papilla regeneration, and explored the potential correlation between LED light and papilla regeneration in relation to this pathway. Our results demonstrate for the first time that the Notch signaling pathway is involved in papilla regeneration in *A. japonicus*, and preliminarily reveal the expression characteristics of the pathway regulated by LED light intensity.

## 2. Results

### 2.1. Observation of Morphological Characteristics, Gene Expression Analysis and Cell Proliferation Detection During Papilla Regeneration

**Figure 1:**
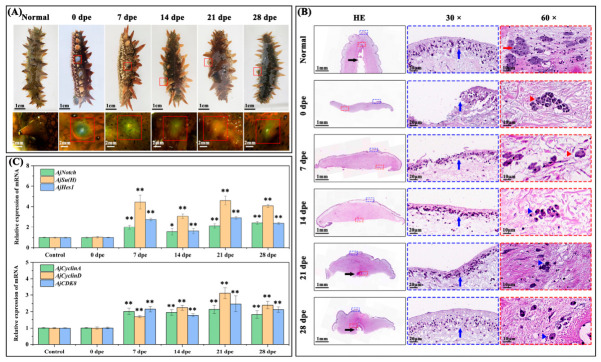
Morphological observation, histological analysis, and gene expression profiles during papilla regeneration in *A. japonicus*. (**A**) Morphological observation of papilla regeneration in *A. japonicus* at different time points post excision. Upper panels: gross morphology of intact papillae and papillary excision sites, scale bar = 1 cm. Lower panels: magnified views of intact papillae and papillary excision sites, scale bar = 2 mm. Red boxes indicate the excision site. (**B**) HE staining of regenerating papillae in *A. japonicus* at different time points. Left column: panoramic views of tissue sections, scale bar = 1 mm. Middle column (30× magnification): enlarged views of the blue dashed box region, scale bar = 20 μm. Right column (60× magnification): enlarged views of the red dashed box region, scale bar = 10 μm. Black arrows indicate the water vascular cavity, blue arrows indicate epidermal cells, red arrows indicate mesenchymal-like cells. Red triangles denote morula-like cells, and blue triangles denote mononuclear cells. (**C**) Relative expression levels of Notch signaling pathway genes (*AjNotch*, *AjSu(H)*, and *AjHes1*) and cell cycle-related genes (*AjCyclinA*, *AjCyclinD*, and *AjCDK8*) during papilla regeneration in *A. japonicus*. Data are presented as the mean from three replicate groups with three sea cucumber samples per group (N = 9). * *p* < 0.05, ** *p* < 0.01, compared with the control group (intact normal papillae). Error bars represent standard deviation (SD).

To clarify the regeneration process of *A. japonicus* papillae after excision, continuous morphological observations were conducted over a 28-day regeneration period. At 7 days post papilla excision (dpe), microscopic observations revealed that the papillary incision site was nearly healed, and scar-like structures with irregular pigment streaks were visible at some healed areas in partial individuals. HE staining revealed morphological changes consistent with the dedifferentiation of mature mesenchymal-like cells in the connective tissue of regenerating papillae, as evidenced by the formation of multinucleated morula-like structures. Meanwhile, cells in the surrounding tissues were observed to accumulate toward the epidermal layer and aggregate to form an initial single- to double-layered cellular structure (Figure 1A,B). At 14 dpe, microscopic observations revealed accelerated regeneration at the papillary excision site, with the formation of an initial protuberance at the papillary base and the scar-like structures with irregular pigment streaks observed at 7 dpe having gradually disappeared. HE staining showed a continuous increase in the number of epidermal cells in the regenerating papillae, which formed an irregularly arranged multi-layered cellular structure. In the connective tissue, the morula-like cells were observed to undergo morphological changes and give rise to a large number of mononuclear cells (Figure 1A,B). By 28 dpe, microscopic examination revealed a slow increase in the length of regenerating papillae, and most developed a protuberance at the base of the papillary excision site. HE staining analysis showed that epidermal cells in the regenerating papillae were arranged regularly in 2–3 layers, and an initial water vascular cavity was formed in the papilla tissues (Figure 1A,B). Gene expression analysis showed that the relative expression levels of three Notch signaling pathway-related genes (*AjNotch*, *AjSu(H)*, and *AjHes1*) were significantly upregulated at 7, 14, 21, and 28 dpe compared with the control group (intact normal papillae) (*p* < 0.05) (Figure 1C). The peak expression levels of *AjSu(H)* and *AjHes1* were observed at 21 dpe, while *AjNotch* reached its peak at 28 dpe, showing extremely significant increases of 4.60-fold, 2.92-fold, and 2.42-fold, respectively (*p* < 0.01) (Figure 1C). In addition, compared with the control group, the relative expression levels of three cell proliferation-related genes (*AjCyclinA*, *AjCyclinD*, and *AjCDK8*) were significantly upregulated at 7, 14, 21, and 28 dpe. The peak relative expression levels of *AjCyclinA*, *AjCyclinD*, and *AjCDK8* all appeared at 21 dpe, exhibiting extremely significant increases of 2.14-fold, 3.12-fold, and 2.47-fold, respectively (*p* < 0.01) (Figure 1C).

**Figure 2:**
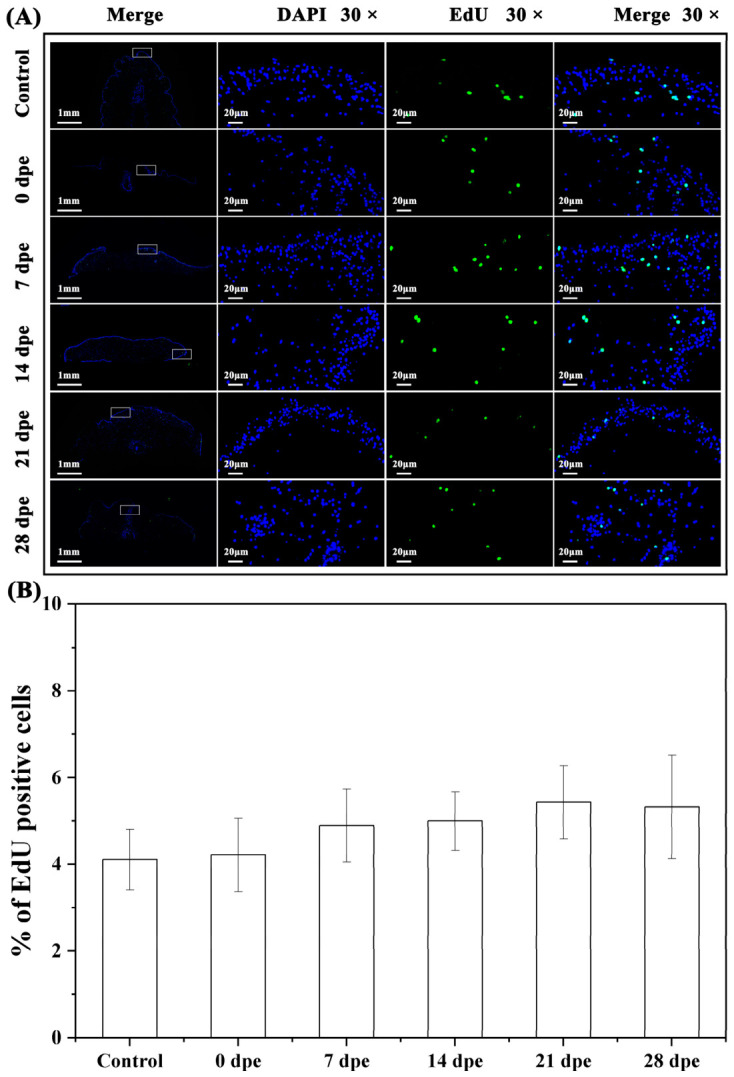
Cell proliferation during papilla regeneration in *A. japonicus* at different time points post excision. (**A**) Tissue distribution of EdU-positive signals during papilla regeneration. The first column on the left shows the panoramic view of tissue EdU staining, and the three columns on the right display the 30× magnified images of the tissues within the white boxes. Full tissue views are shown with a scale bar of 1 mm, and magnified tissue regions are marked with a scale bar of 20 μm. (**B**) Proportion of EdU-positive cells during papilla regeneration in *A. japonicus* at different time points post excision. Data are presented as the mean from three replicate groups with three sea cucumber samples per group (N = 9). Error bars represent standard deviation (SD).

EdU staining results showed an increasing trend in the proportion of EdU-positive signal-labeled cells during *A. japonicus* papilla regeneration. The highest proportion (5.44 ± 0.84%) was observed at 21 dpe, while the lowest proportion (4.11 ± 0.69%) was detected in the control group. No significant differences were observed in the proportion of EdU-positive signal-labeled cells at 0, 7, 14, 21, and 28 dpe compared with that in the control group (*p* > 0.05) (Figure 2A,B).

### 2.2. Effects of Different Light Intensity on the Regenerating Papilla Growth and Development

**Figure 3:**
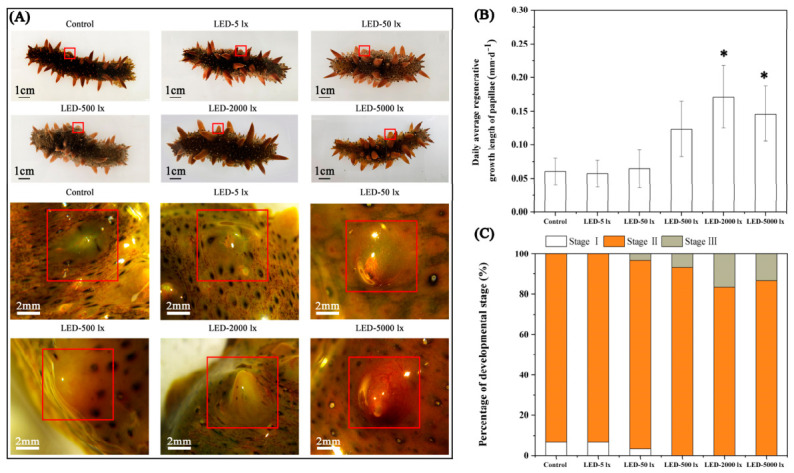
Effects of different light intensity conditions on papilla regeneration rate in *A. japonicus*. (**A**) Morphological observation of regenerating papillae in *A. japonicus* under different light intensity conditions. Upper panels: gross morphology of regenerating papillae under control and LED light treatments (5, 50, 500, 2000, and 5000 lx), scale bar = 1 cm. Lower panels: magnified views of regenerating papillae under the corresponding light conditions, scale bar = 2 mm. Red boxes indicate the regenerating papilla region. (**B**) Daily average regenerative growth length of papillae in *A. japonicus* under different light intensity conditions. (**C**) Proportion of *A. japonicus* individuals at different developmental stages of regenerating papillae under different light intensity conditions. The three stages were defined as: Stage I, the papillary wound is incompletely healed or remains flat after healing; Stage II, the wound is fully healed with a visible protrusion formed; Stage III, the papilla structure is well-developed with a papillary length to body length ratio (PL/BL) > 0.05. Data are presented as the mean from three replicate groups with three sea cucumber samples per group (N = 30). * significant difference at *p* < 0.05 vs. Control. Error bars represent standard deviation (SD).

To explore the influence of LED light on papilla regeneration in *A. japonicus*, we assessed the regenerative development rate and morphological structural changes in its papillae at varying light intensities. After 28 days of papilla regeneration, the average daily regeneration rate of sea cucumber papillae under 2000 lx and 5000 lx light intensities was significantly higher than that of the dark control group (0.06 ± 0.02 mm·d^−1^) (*p* < 0.05). The sea cucumbers exposed to 2000 lx LED light exhibited the fastest growth of regenerating papillae, with an average daily regeneration rate of 0.17 ± 0.05 mm·d^−1^ (Figure 3A,B). After 28 days of papilla regeneration, a subset of sea cucumbers in the dark control group (6.67%), LED-5 lx irradiation group (6.67%), and LED-50 lx light group (3.33%) had incompletely healed wounds, while all individuals in the LED-500/2000/5000 lx groups exhibited full wound healing (Figure 3C).

**Figure 4:**
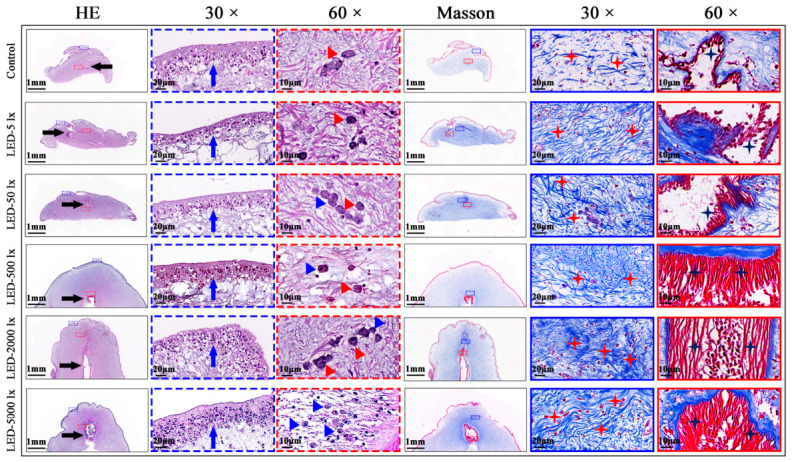
Effects of different light intensities on the histological structure of regenerating papillae in *A. japonicus*. HE staining (left three columns) and Masson staining (right three columns) were performed on regenerating papillae at 28 days post regeneration. Panoramic views (first column of each staining set): Observed under a 2× objective lens, scale bar = 1 mm. Black arrows indicate the water vascular cavity. Images of 30× magnification views (second column of each staining set): Enlarged views of the blue dashed box regions (HE) and solid blue box regions (Masson), scale bar = 20 μm. For HE staining, blue arrows indicate epidermal cells; for Masson staining, red asterisks indicate the distribution of collagen fibers. Images of 60× magnification views (third column of each staining set): Enlarged views of the red dashed box regions (HE) and solid red box regions (Masson), scale bar = 10 μm. For HE staining, red triangles denote morula-like cells and blue triangles denote mononuclear cells; for Masson staining, blue asterisks indicate the distribution of muscle fibers.

After 28 days of papilla regeneration, HE staining results showed that with increasing light intensity, the number of epidermal cell layers in regenerating papillae, the proliferation and differentiation levels of morula-like cells in the connective tissue, and the differentiation degree of water vascular cavity structures in each experimental group all exhibited an increasing trend. Meanwhile, the distribution of epidermal cells and morula-like cells in regenerating papillae became more uniform as light intensity increased; compared with the control group, the LED-2000 lx group showed the most prominent differentiation of the water vascular cavity (Figure 4). Masson staining results indicated that increasing light intensity promoted the formation of collagen fibers in the connective tissue and muscle fibers within the water vascular cavity structures during papilla regeneration. Compared with the control group, the LED-2000 lx group displayed the most concentrated collagen fiber distribution and a more mature state of muscle fiber differentiation (Figure 4).

### 2.3. Effects of Different Light Intensity on Gene Expression and Cell Proliferation During Papilla Regeneration

**Figure 5:**
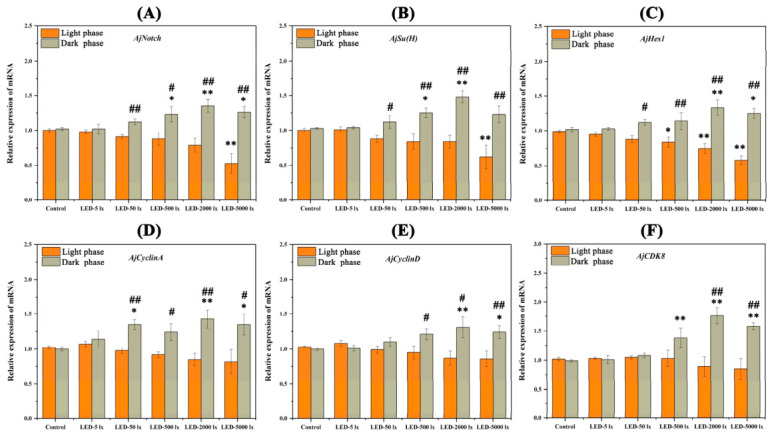
Effects of different light intensities on gene expression during papilla regeneration in *A. japonicus*. (**A**–**C**) Relative expression levels of *AjNotch*, *AjSu(H)*, and *AjHes1.* (**D**–**F**) Relative expression levels of *AjCyclinA*, *AjCyclinD*, and *AjCDK8*. Data are presented as the mean from three replicate groups with three sea cucumber samples per group (N = 9). Different colored columns represent the light phase and dark phase, respectively. * significant difference at *p* < 0.05 vs. Control, ** extremely significant difference at *p* < 0.01 vs. Control; # significant difference at *p* < 0.05 vs. the light phase, ## extremely significant difference at *p* < 0.01 vs. the light phase. Error bars represent standard deviation (SD).

Gene expression and cell proliferation were compared among light intensity groups and between light/dark phases. Dual-phase sampling was used to account for circadian effects on gene expression. To investigate the effects of LED light on papilla regeneration in *A. japonicus*, we assessed the relative expression levels of genes in the Notch signaling pathway and its downstream cell proliferation-related genes during regeneration. After 28 days of papilla regeneration, the relative expression levels of three key Notch signaling pathway genes (*AjNotch*, *AjSu(H)*, and *AjHes1*) gradually decreased with the increase in light intensity in the light phase (Figure 5A–C). Specifically, compared with the control group, the expression levels of *AjNotch* and *AjSu(H)* were extremely significantly downregulated in the 5000 lx LED treatment group (*p* < 0.01), whereas those of *AjHes1* were significantly downregulated in the 500, 2000, and 5000 lx LED treatment groups (*p* < 0.05) (Figure 5A–C). After 28 days of papilla regeneration, the relative expression of three key genes in the Notch signaling pathway exhibited an increasing trend with rising light intensity in the dark phase (Figure 5A–C). Specifically, compared with the control group, *AjNotch* expression was significantly upregulated in the 500, 2000, and 5000 lx LED treatment groups, whereas *AjSu(H)* expression was significantly upregulated in the 500 and 2000 lx LED treatment groups, and *AjHes1* expression was significantly upregulated in the 2000 and 5000 lx LED treatment groups (*p* < 0.05) (Figure 5A–C). Compared within the light phase, the relative expression of *AjNotch*, *AjSu(H)* and *AjHes1* was significantly upregulated in the 50, 500, 2000, and 5000 lx LED groups in the dark phase (*p* < 0.05) (Figure 5A–C). After 28 days of papilla regeneration, the relative expression of three key cell proliferation-related genes (*AjCyclinA*, *AjCyclinD* and *AjCDK8*) decreased with elevated light intensity in the light phase (*p* > 0.05) (Figure 5D–F). By contrast, the relative expression levels of these three genes showed an upward trend with the increase in light intensity at 28 dpe. Specifically, compared with the control group, the relative expression of *AjCyclinA* was significantly upregulated in the 50, 2000, and 5000 lx LED treatment groups (*p* < 0.05), that of *AjCyclinD* was significantly upregulated in the 2000 and 5000 lx LED treatment groups (*p* < 0.05), and that of *AjCDK8* was extremely significantly upregulated in the 500, 2000, and 5000 lx LED treatment groups (*p* < 0.01) (Figure 5D–F). Compared within the light phase, the relative expression of *AjCyclinA* was significantly upregulated in the 50, 500, 2000 and 5000 lx LED groups in the dark phase (*p* < 0.05), that of *AjCyclinD* was significantly upregulated in the 500, 2000, and 5000 lx LED groups in the dark phase (*p* < 0.05), and that of *AjCDK8* was significantly upregulated in the 2000 and 5000 lx LED groups in the dark phase (*p* < 0.05) (Figure 5D–F).

**Figure 6:**
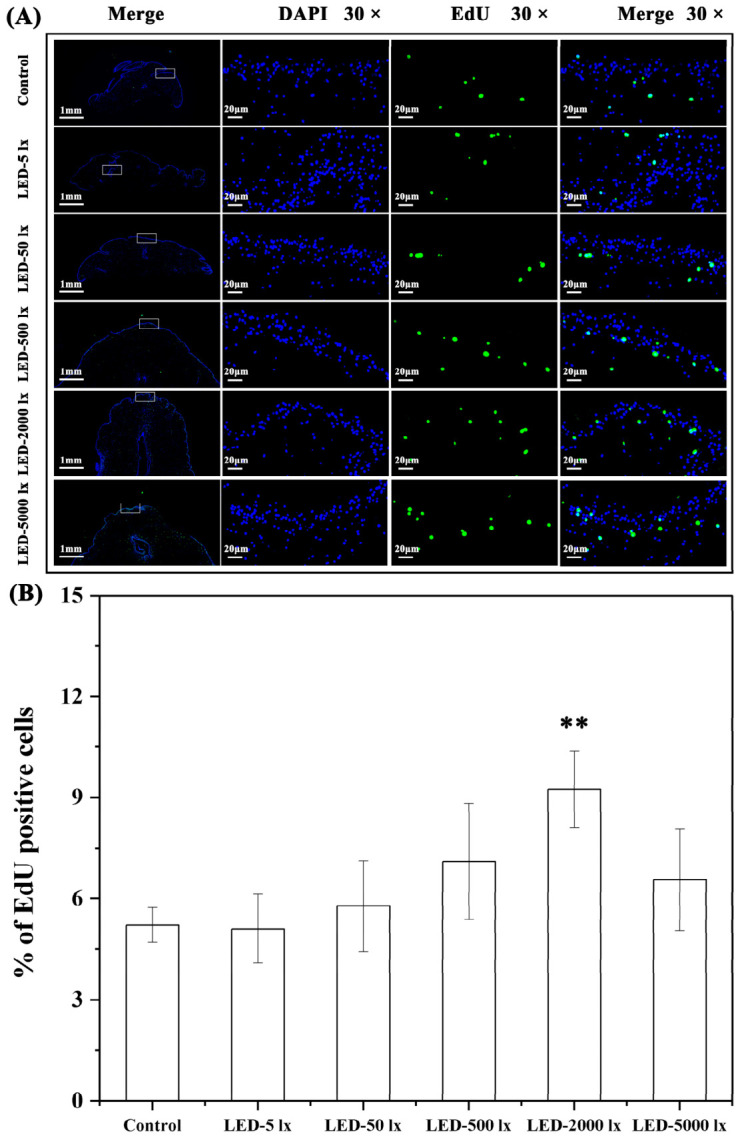
Effects of different light intensity conditions on cell proliferation during papilla regeneration in *A. japonicus*. (**A**) Tissue distribution of EdU-positive signals during papilla regeneration in *A. japonicus*. The first column on the left shows the panoramic view of tissue EdU staining, and the three columns on the right display the 30× magnified images of the tissues within the white boxes. Full tissue views are shown with a scale bar of 1 mm, and magnified tissue regions are marked with a scale bar of 20 μm. (**B**) Proportion of EdU-positive cells during papilla regeneration in *A. japonicus*. Data are presented as the mean from three replicate groups with three sea cucumber samples per group (N = 9). ** extremely significant difference at *p* < 0.01 vs. Control. Error bars represent standard deviation (SD).

After 28 days of papilla regeneration, EdU staining assays showed that there was a positive correlation between light intensity and the proportion of EdU-positive cells in each LED group (Figure 6A,B). Specifically, the LED-2000 lx group exhibited the highest proportion of EdU-positive cells (9.24 ± 1.14%), whereas the LED-5 lx group had the lowest (5.11 ± 1.02%). Notably, the proportion of EdU-positive cells in the LED-2000 lx group differed significantly from that in the control group (*p* < 0.01) (Figure 6B).

### 2.4. Silenced AjNotch Affected Downstream Target Gene Expression and Cell Proliferation During the Papilla Regeneration

**Figure 7:**
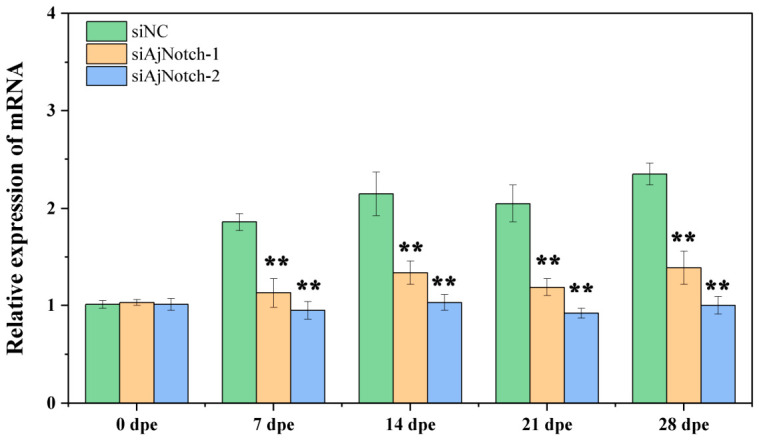
Effects of gene silencing on the relative expression level of *AjNotch* during papilla regeneration in *A. japonicus* at different time points. Data are presented as the mean from three replicate groups with three sea cucumber samples per group (N = 9). ** extremely significant difference at *p* < 0.01 vs. siNC. Error bars represent standard deviation (SD).

To further investigate the role of *AjNotch* in *A. japonicus* papilla regeneration, the relative expression level of *AjNotch* was detected after treatment with siAjNotch-1, siAjNotch-2, or siNC (negative control). In the siAjNotch-1 group, the relative expression level of *AjNotch* decreased to approximately 61%, 62%, 58%, and 59% of that in the siNC group at 7, 14, 21, and 28 dpe, respectively, with an average interference efficiency exceeding 40% (*p* < 0.05) (Figure 7). In the siAjNotch-2 group, the relative expression level of *AjNotch* decreased to approximately 51%, 48%, 45%, and 43% of that in the siNC group at 7, 14, 21, and 28 dpe, respectively, with an average interference efficiency exceeding 53% (*p* < 0.05) (Figure 7). These results indicated that both siAjNotch-1 and siAjNotch-2 significantly reduced the relative expression level of the target gene *AjNotch* over 28 days of continuous interference (*p* < 0.05) (Figure 7). Notably, siAjNotch-2 showed a higher average interference efficiency against *AjNotch* than siAjNotch-1.

**Figure 8:**
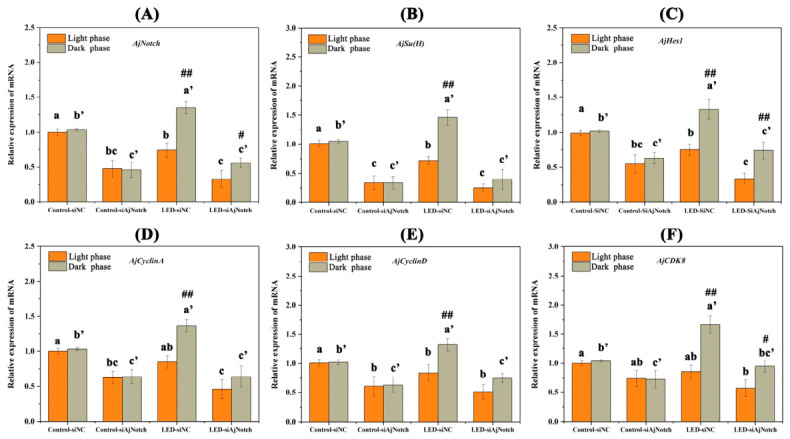
Effects of different light intensities on gene expression during papilla regeneration in *A. japonicus* following gene silencing. (**A**–**C**) Relative expression levels of *AjNotch*, *AjSu(H)*, and *AjHes1*. (**D**–**F**) Relative expression levels of *AjCyclinA*, *AjCyclinD*, and *AjCDK8*. Data are presented as the mean from three replicate groups with three sea cucumber samples per group (N = 9). Different colored columns represent the light phase and dark phase, respectively. Lowercase letters denote intergroup differences in the light phase (*p* < 0.05), and primed letters denote intergroup differences in the dark phas (*p* < 0.05). # significant difference at *p* < 0.05 vs. the light phase, ## extremely significant difference at *p* < 0.01 vs. the light phase. Error bars represent standard deviation (SD).

Gene expression and cell proliferation were compared between control and LED groups and between light/dark phases. Samples were collected in both phases to eliminate circadian interference. SiAjNotch-2 was selected to further investigate the expression of downstream target genes of the Notch signaling pathway and cell proliferation-related genes. After 28 days of continuous interference with *AjNotch*, in the light phase, the relative expression levels of *AjNotch*, *AjSu(H)*, and *AjHes1* in the LED-siNC group were significantly lower than those in the Control-siNC group (*p* < 0.05) (Figure 8A–C). In contrast, in the dark phase, the relative expression levels of *AjNotch*, *AjSu(H)*, and *AjHes1* in the LED-siNC group were significantly higher than those in the Control-siNC group (*p* < 0.05) (Figure 8A–C). After 28 days of continuous interference with *AjNotch*, the relative expression levels of *AjNotch*, *AjSu(H)*, and *AjHes1* in both the Control and LED groups were significantly reduced in both the light phase and dark phase (*p* < 0.05) (Figure 8A–C). Additionally, significant differences were observed in the relative expression levels of *AjNotch*, *AjSu(H)*, and *AjHes1* in the LED-siNC group between the light phase and the dark phase (*p* < 0.05) (Figure 8A–C). After 28 days of continuous interference with *AjNotch*, in the light phase, the relative expression level of *AjCyclinD* in the LED-siNC group was significantly lower than that in the Control-siNC group (*p* < 0.05) (Figure 8E). In contrast, in the dark phase, the relative expression levels of *AjCyclinA*, *AjCyclinD*, and *AjCDK8* in the LED-siNC group were significantly higher than those in the Control-siNC group (*p* < 0.05) (Figure 8D–F). After 28 days of continuous interference with *AjNotch*, in the light phase, the relative expression levels of *AjCyclinA* and *AjCyclinD* in the Control-siAjNotch group were significantly decreased compared with the Control-siNC group (*p* < 0.05) (Figure 8D,E). In the dark phase, the relative expression levels of *AjCyclinA*, *AjCyclinD*, and *AjCDK8* in the Control-siAjNotch group were also significantly reduced compared with the Control-siNC group (*p* < 0.05) (Figure 8D–F). After 28 days of continuous interference with *AjNotch*, in the light phase, the relative expression level of *AjCyclinA* in the LED-siAjNotch group was significantly lower than that in the LED-siNC group (*p* < 0.05) (Figure 8D). In contrast, in the dark phase, the relative expression levels of *AjCyclinA*, *AjCyclinD*, and *AjCDK8* in the LED-siAjNotch group were significantly decreased compared with the LED-siNC group (*p* < 0.05) (Figure 8D–F).

**Figure 9:**
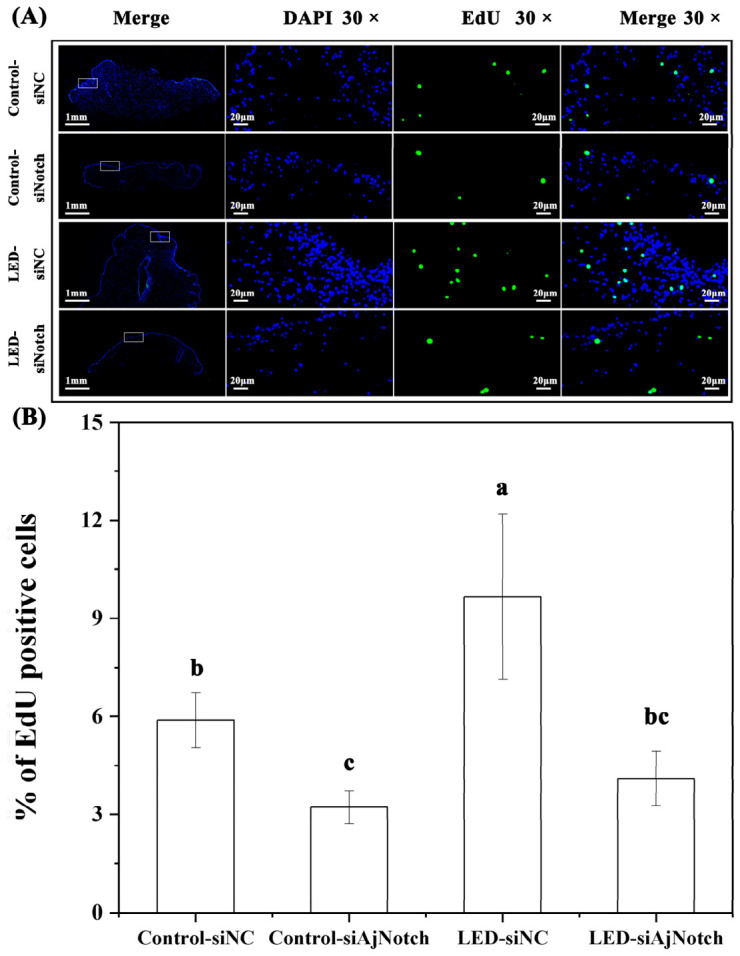
Effects of different light intensities on cell proliferation during papilla regeneration in *A. japonicus* following gene silencing. (**A**) Tissue distribution of EdU-positive signals in regenerating papillae. The first column on the left shows the panoramic view of tissue EdU staining, and the three columns on the right display the 30× magnified images of the tissues within the white boxes. Full tissue views are shown with a scale bar of 1 mm, and magnified tissue regions are marked with a scale bar of 20 μm. (**B**) Proportion of EdU-positive cells in regenerating papillae. Data are presented as the mean from three replicate groups with three sea cucumber samples per group (N = 9). Different letters indicate a significant difference. Error bars represent standard deviation (SD).

After 28 days of continuous interference with *AjNotch*, EdU staining results showed that the proportion of EdU-positive cells in the LED-siNC group (9.67 ± 2.52%) was significantly higher than that in the Control-siNC group (5.89 ± 0.84%) (*p* < 0.05) (Figure 9A,B). After 28 days of continuous interference with *AjNotch*, the proportion of EdU-positive cells in both the Control and LED groups was significantly decreased (*p* < 0.05) (Figure 9A,B).

## 3. Discussion

Morphological observation, as a fundamental yet crucial technique in regenerative biology, has been widely applied in investigating the regeneration of tissues and organs in echinoderms, including internal organs, body walls, and lateral appendages [10,11,32]. This approach serves as a core entry point for analyzing the spatiotemporal patterns of regeneration in the sea cucumber *A. japonicus* [44]. In the present study, microscopic observations and HE staining demonstrated that wound healing and structural regeneration of excised papillae were largely accomplished within 28 days, although full morphological maturation was not fully achieved. During the early stage of regeneration (0–7 dpe), rapid healing of the papillary incision site was observed, accompanied by the formation of scar-like structures. Notably, wound healing in the sea cucumber body wall typically completes within a few days; for example, Jiao et al. (2024) reported that wound closure could be achieved within 3 days after excision [45]. On the 7th day post regeneration, irregular scar-like streaks were observed at the healed sites of the papilla in some sea cucumbers. The superficial layer of the sea cucumber body wall is generally rich in melanocytes, which are closely clustered and may function to reduce light exposure and mitigate ultraviolet (UV) damage [46,47]. Based on these observations, we propose that the early stages of papilla regeneration in *A. japonicus* share striking similarities with the body wall regeneration process: cells within the tissues adjacent to the papillary wound migrate rapidly toward the epidermal surface, and melanocyte-like cells generated via cellular proliferation exhibit irregular arrangement, which in turn leads to the formation of scar-like structures on the wound surface. From our observations, we suggest that 7–14 dpe constitutes a critical period for papillary primordium formation, during which the water vascular cavity enters its initial developmental stage. Previous studies have shown that in echinoderm regeneration, the water vascular system is one of the first organs to recover, laying a structural foundation for appendage regeneration [48,49]. In contrast to sea cucumber body wall regeneration, the obvious differentiation of the water vascular cavity during papilla regeneration is a distinctive characteristic and may serve as a key indicator of papillary regeneration. More importantly, this differentiation of the water vascular cavity may be the primary driving force underlying the outward extension and growth of regenerating papillae.

During the embryonic development of echinoderms, the Notch signaling pathway plays a crucial role in embryogenesis, tissue formation, and organ primordium construction by coordinating with other signaling pathways [24,32,50]. Its functions have been well validated in echinoderm species such as sea urchins and starfish [24]. Our findings showed that *AjNotch*, *AjSu(H)*, and *AjHes1* were significantly upregulated at 7, 14, 21, and 28 dpe, suggesting that the Notch signaling pathway is involved in the positive regulation of *A. japonicus* papilla regeneration. This result is consistent with the research of Mashanov et al. (2022), who confirmed that activation of the Notch signaling pathway is essential for wrist regeneration in *O. brevispina* and also participates in cell migration, matrix remodeling, and immune clearance processes during regeneration [32]. In the present study, the expression levels of *AjNotch*, *AjSu(H)*, and *AjHes1* exhibited oscillatory upregulation during papilla regeneration, with peak expressions observed at 7 and 21 dpe, respectively, followed by subsequent downregulation. As crucial downstream effectors of the Notch signaling pathway, members of the Hes family precisely regulate the expression of cyclin-dependent kinase inhibitors (CKIs) and cyclins, thereby balancing the cellular states of proliferation, quiescence, and differentiation. During these phases, the Notch signaling pathway may effectively regulate the dynamics of cell proliferation and differentiation through the oscillatory expression of *AjHes1*, thereby maintaining the ordered progression of papilla regeneration [28,51]. Cyclins and cyclin-dependent kinases (CDKs) play a pivotal role in the precise regulation of the cell cycle [42,43]. Our study revealed a consistent expression pattern between three cell proliferation-related genes and key genes of the Notch signaling pathway, indicating that *AjCyclinA*, *AjCyclinD*, and *AjCDK8* may serve as critical downstream target genes of the Notch signaling pathway. Previous research on *Eriocheir sinensis* (Chinese mitten crab) demonstrated that the Notch signaling pathway regulates the cell proliferation-associated gene *CDK1* through two target genes, *Hes 1* and *Hey L*. Additionally, *CyclinA* and *CyclinE* have been shown to be involved in regulating the regeneration process in *E. sinensis*, which further supports the potential regulatory link between the Notch signaling pathway and cell cycle-related genes in invertebrate regeneration [52].

Studies have established that appropriate light stimulation can promote wound healing, tissue repair, and organ regeneration in marine organisms [36,37]. Early research has demonstrated that light intensity exerts a significant regulatory effect on the growth, development, and feeding activity of echinoderms [38,53]. However, research focusing on the effects of light intensity on wound healing and tissue regeneration in echinoderms remains extremely limited. The present study demonstrated that the LED light significantly promotes the regeneration of *A. japonicus* papillae. Specifically, the average daily regeneration rate of papillae in the LED-2000 lx group was approximately three times higher than that in the all-dark control group, which underscores the strong regenerative plasticity of sea cucumber papillae. HE and Masson’s trichrome staining results revealed that continuous LED light stimulation significantly promoted cell dedifferentiation, proliferation, and differentiation in the regenerating papilla tissues of *A. japonicus*. Meanwhile, the density of collagen fibers and muscle fibers were notably increased. These morphological changes suggest that LED light may regulate papilla regeneration by modulating cell proliferation and differentiation at the regenerative site. Mesenchymal-like cells were frequently observed in close association with collagen fibers, implying that these cells may represent fibroblasts or fibroblast-like cells. As the main cellular component of connective tissue, fibroblasts are generally thought to participate in extracellular matrix synthesis and tissue repair [54,55]. During papilla regeneration, such cells may contribute to collagen production and extracellular matrix remodeling, thereby supporting tissue reconstruction and structural integrity. Taken together, these histological observations indicate that LED light may facilitate the structural maturation of regenerating papillae by promoting cell proliferation, differentiation, and extracellular matrix deposition. However, the precise cellular sources and regulatory mechanisms underlying these processes require further investigation.

Studies have demonstrated that LED light can enhance cellular mitochondrial function, thereby promoting increased ATP production and providing additional energy for cell proliferation [56,57,58]. For instance, when the wound surface of *Paralichthys olivaceus* (olive flounder) was exposed to green LED light, the 40 μmol·m^−2^·s^−1^ group (a moderate light intensity used in a previous wound healing study) exhibited a significant increase in the number of proliferating cells, which was associated with the fastest wound healing rate [37]. To assess the potential molecular correlates of papilla regeneration in *A. japonicus* under varying light intensities, we analyzed the expression patterns of *AjNotch* and its downstream target genes. Our results showed that during sea cucumber papilla regeneration, the expression levels of these genes exhibited a downward trend in the light phase, whereas an upward trend was observed in the dark phase. Sea cucumbers exhibit a distinct circadian rhythm, with opsins and clock genes serving as key regulatory factors for their physiological activities and behaviors [38,59]. Opsins are localized in sensory cells such as tentacles, papillae, and tube feet [38]. Existing studies have shown that opsins can transmit light signals via the cAMP-PKA-ERK pathway, and PKA-ERK can form a complex mutually regulatory network with Notch signaling, participating in various physiological processes including tissue regeneration [38,60,61]. Additionally, opsin activation significantly reduces intracellular cAMP levels [38], suggesting that LED light may regulate cAMP levels through an opsin-mediated phototransduction system, thereby influencing the transduction of the Notch signaling pathway. However, this study only identified a correlation between light regimes and the oscillatory expression of Notch signaling, and could not confirm direct transduction via the opsin–cAMP–PKA–ERK cascade. The interaction between the endogenous circadian clock of sea cucumbers and exogenous light cycles remains unclear, and the fixed photoperiod and sampling time in this study may not be optimal. Since circadian rhythms strongly affect gene expression and light sensitivity, testing different photoperiods and time-resolved sampling is expected to better reveal the dynamic crosstalk among light input, the internal clock, and Notch signaling. Future studies with systematic photoperiod gradients and refined temporal sampling will help define the optimal light pattern and clarify the molecular link between light and papilla regeneration.

Notch signaling is a highly conserved core pathway in echinoderms, with critical roles in embryonic development and tissue regeneration widely documented across species. In sea urchins, it is essential for endomesoderm segregation and secondary mesenchyme cell specification [50]. In the brittle star *O. brevispinum*, normal arm regeneration relies on active Notch signaling, and multiple related genes have been identified in its genome, supporting further research on regeneration [32,62]. Numerous studies have shown that the Notch signaling pathway regulates stem cell proliferation and differentiation through the oscillatory expression of *Hes1* [63,64]. In the present study, although increasing light intensity in the light phase was associated with a slight reduction in the expression levels of Notch signaling pathway–related genes, it did not significantly decrease the relative expression of downstream cell cycle–related genes, including *AjCyclinA*, *AjCyclinD*, and *AjCDK8*. In contrast, in the dark phase, the relative expression levels of these three proliferation-related genes were significantly upregulated in both the LED-2000 lx and LED-5000 lx groups. This higher gene expression in the dark phase may be attributed to the nocturnal nature of *A. japonicus*, which is more physiologically active in darkness [34,38,59]. Notably, the EdU incorporation data reflect the cumulative effect of cell proliferation after both light and dark treatments rather than proliferation status under a single condition alone (Figure 6). The overall higher cell proliferation signal observed in EdU staining may therefore represent a combined effect of the non-significantly reduced gene expression during the light phase and the markedly enhanced gene expression during the dark phase. This cumulative effect could reconcile the seemingly inconsistent trends between short-term gene expression changes and the overall proliferation activity reflected by EdU labeling. Together, these results suggest that periodic light–dark alternation may be more favorable for papilla regeneration and development than continuous light exposure.

EdU staining results showed that the proportion of EdU-positive proliferating cells in the LED-5000 lx group was higher than that in the complete dark control group, yet lower than that in the LED-2000 lx group. Meanwhile, the relative expression levels of three key genes in the Notch signaling pathway (*AjNotch*, *AjSu(H*) and *AjHes1*) in the LED-5000 lx group were all significantly downregulated compared with the control group. Integrating these results, we consider that stimulation with an appropriate light intensity can enhance the regeneration and development of sea cucumber papillae, whereas excessively high light intensity may impede papilla regeneration by inhibiting cell proliferation. Therefore, we propose that LED light at 2000 lx is the optimal condition for sea cucumber papilla regeneration.

The Notch signaling pathway plays a crucial role in regulating embryonic development, cell differentiation, and tissue regeneration in echinoderms, and can be used in functional experiments to specifically validate the regulatory roles of target genes [24,32]. As a conserved core signaling pathway in echinoderm regeneration, Notch signaling has been confirmed to be indispensable for the normal regeneration of multiple appendages in different echinoderm species: inhibition of Notch signaling by DAPT, a specific pathway inhibitor, can significantly block arm regeneration in the brittle star *O. brevispinum*, and lead to abnormal cell proliferation and pattern reconstruction at the regeneration site, while genomic analysis of this species has also identified a complete set of Notch pathway-related genes, further supporting the conserved regulatory function of this pathway in echinoderm regeneration [32,50]. Our gene silencing experiment demonstrated that sustained interference with *AjNotch* expression significantly downregulated its downstream target genes *AjSu(H)* and *AjHes1*, and markedly reduced the cell proliferation rate at the papilla regeneration site of *A. japonicus*. This result is consistent with findings in the Chinese mitten crab, where Notch signaling modulates appendage regeneration via *Hes1* and *HeyL*, and further corroborates the evolutionary conservation of Notch signaling in regulating invertebrate appendage regeneration [28,30,52]. However, due to the limitations of the coelomic injection method for delivering the interference reagent, we only achieved moderate knockdown efficiency in this study, meaning a considerable proportion of Notch protein may retain its biological activity. Future investigations should further evaluate the silencing efficiency at the protein level for more robust validation.

Notably, our LED light irradiation experiments not only identified 2000 lx as the optimal light intensity for promoting papilla regeneration in *A. japonicus*, but also implied the potential regulatory value of light cycles: the periodic alternation of light and dark adopted in this experiment can induce oscillatory expression of Notch pathway-related genes and their downstream cell cycle-related genes, which is consistent with the conserved dynamic regulatory characteristics of Notch signaling during development [41,63,64]. Based on existing research reports, it is speculated that this oscillatory mode, compared with sustained activation, may be more conducive to balancing the maintenance and directed differentiation of progenitor cells during regeneration, and providing necessary regulatory signals for the ordered regeneration of *A. japonicus* papillae. However, this study still has certain limitations: On the one hand, only a single light cycle regime was investigated; thus, the generality of its regulatory effect on Notch signal oscillation, as well as the molecular relationship between light signal transduction and Notch oscillation, remains to be further elucidated. On the other hand, the specificity of gene interference and the dynamic characteristics of oscillatory gene expression at the single-cell level have not been fully verified. These limitations indicate that further in-depth investigation is still required to systematically reveal the molecular mechanism by which light regulates papilla regeneration in *A. japonicus*.

## 4. Materials and Methods

### 4.1. Animals and Treatment

Sea cucumbers (*A. japonicus*) were purchased from Yinhaima Aquatic Products Breeding (Dalian) Co., Ltd. (Dalian, China). A total of 500 healthy individuals with an average body weight of 25.35 ± 5.23 g were reared in the Key Laboratory of Mariculture & Stock Enhancement in the North China Sea, Ministry of Agriculture, Dalian Ocean University, Dalian, China. During the acclimation period, seawater temperature was maintained at 15–17 °C, with daily water exchange. Sea cucumbers were fed once daily with a commercial formulated diet for sea cucumber, at a rate of approximately 3% of their body weight per day. Acclimation was performed under natural light conditions throughout the experiment.

### 4.2. RNA Extraction and Gene Expression Analysis

Papilla tissues from both normal and regenerating *A. japonicus* were selected for total RNA extraction. Total RNA of the selected tissues was extracted using the RNAprep Pure Tissue Kit (Tiangen, Beijing, China) according to the manufacturer’s instructions. The quality and quantity of total RNA were detected using 1% agarose gel electrophoresis and UV spectrophotometry, respectively; UV spectrophotometry was performed on a NanoPhotometer (Implen, München, Germany).

First-strand cDNA was synthesized from total RNA by reverse transcription using PrimeScript™ RT Master Mix (Takara, Shiga, Japan). The expression levels of six genes (*AjNotch*, *AjSu(H)*, *AjHes1*, *AjCyclinA*, *AjCyclinD*, and *AjCDK8*) were detected using the Applied Biosystems 7500 Real-Time PCR System (Applied Biosystems, Foster City, CA, USA). Primers were designed with Primer Premier 5.0 software, and all sequences are given in Table 1. *Cytochrome b* (*Cytb*) was used as the internal reference gene for normalization (Table 1). The qRT-PCR reaction was carried out in a 20 μL volume containing 2 μL of cDNA template, 10 μL of 2 × SYBR Green Master Mix (Takara, Shiga, Japan), 0.4 μL of ROX Reference Dye II, 6 μL of PCR-grade water, and 0.8 μL (10 mM) of each forward and reverse primer. Three biological replicates with three technical replicates each were set for each gene, and the mean of the three biological replicates was used for subsequent relative quantification analysis. The PCR program was set as follows: initial denaturation at 95 °C for 30 s, followed by 40 cycles of denaturation at 95 °C for 5 s and annealing/extension at 60 °C for 32 s. After amplification, a melting curve analysis was performed to confirm the specificity of the PCR products. The relative expression levels of the target genes were calculated using the 2^−ΔΔCt^ method [65].

### 4.3. Papilla Regeneration Experiment

Prior to the start of the experiment, a total of 54 healthy sea cucumbers with consistent body size traits were selected following temporary acclimation. After the experimental sea cucumbers were anaesthetized, the ventral papillae of each individual were excised to facilitate subsequent observation and measurement. To ensure uniform wound size after papilla excision, the methodology described by Jiao et al. (2024) was adopted: The wound surface was shaped into a standardized concave form along the body wall direction, with dimensions of 0.5 cm in length, 0.5 cm in width, and 0.2 cm in depth [45].

At the start of the experiment, the 54 sea cucumbers were randomly divided into six groups: five treatment groups with papillae excised and one control group with intact papillae. Each group included three independent biological replicates, with three individuals per replicate. During the entire papilla regeneration period, a constant dark environment was maintained, with daily water exchange and regular feeding performed. Observations of papilla regeneration were conducted on 0, 7, 14, 21, and 28 days post excision (dpe), and tissue samples were collected simultaneously for subsequent staining and gene expression analysis.

### 4.4. LED Light Irradiation Experiment

To explore the effects of LED light (Figure S1) with different intensities on the regeneration and growth of *A. japonicus* papillae, a 28-day experiment was carried out using LED light sources. The experiment included a control group maintained in complete darkness (0 lx) and five experimental groups exposed to different intensities of LED light (LED-5 lx, LED-50 lx, LED-500 lx, LED-2000 lx, and LED-5000 lx).

The light source for the experimental groups was supplied by an E27 LED light device (Shenzhen Well LED Lighting Co., Ltd., Shenzhen, China), with a photoperiod of 12 h light:12 h dark. A total of 360 sea cucumbers were used in the experiment. Following the temporary acclimation rearing phase, healthy sea cucumbers with uniform body size metrics were selected, and the ventral papillae of each sea cucumber were excised in accordance with the protocol detailed in Section 4.3. Each experimental group was set with three independent parallel replicates, with 20 sea cucumbers per replicate. During the regeneration experiment, each replicate of sea cucumbers was reared in 100 L tanks, with daily replacement of fresh seawater and regular feeding maintained throughout the experimental period.

On the 28th day after the experiment initiation, the growth and development of regenerating papillae in all experimental groups of *A. japonicus* were assessed. A total of 30 sea cucumbers were selected for measurement in each experimental group (3 independent parallel groups, 10 individuals per group). Initially, the length of regenerating papillae was measured using a vernier caliper, followed by the evaluation of regeneration progress in each group. The papilla regeneration rate of sea cucumbers (mm·d^−1^) was calculated using the formula: Final length/regeneration days. Based on the observation results, the regeneration and development of papillae were classified into three distinct stages, namely Stage I, Stage II, and Stage III. The determination criteria are as follows: (1) Stage I: The papillary wound is not completely healed or remains flat after healing; (2) Stage II: The papillary wound is completely healed with a protrusion formed; (3) Stage III: The papillary structure is well-defined and meets the minimum biological criterion with a papillary length to body length ratio (PL/BL) greater than 0.05 [19].

On the 28th day of the regeneration experiment, samples were collected at two specific time points to account for diurnal fluctuations: 6 h after the start of the light phase (daytime sampling) and 6 h after the start of the dark phase (nighttime sampling). For each sampling time point, three sea cucumbers were selected from each of three parallel groups to ensure the reliability and statistical significance of the experimental data. The control group, which was kept in constant darkness, was sampled at the same corresponding time points as the light-treated groups to exclude the interference of circadian rhythm on the experimental results. The experimental samples of each group were equally divided into two parts: one part was fixed in 4% paraformaldehyde for subsequent tissue staining and observation, while the other part was quickly frozen in liquid nitrogen and then stored at −80 °C for gene expression analysis.

### 4.5. Silencing of AjNotch Using siRNA

Specific interference oligonucleotides targeting *AjNotch* (siAjNotch-1 and siAjNotch-2) and their negative control siRNA (siNC) were designed and synthesized in GenePharma (Shanghai, China) (Table 2). For in vivo *AjNotch* knockdown, 10 μL of siRNA (20 nM) or siNC was mixed with 10 μL of Lipo2000 transfection reagent (Beyotime, Cat. No. GA5230) and 80 μL of 1× PBS to prepare the transfection solution. At the start of the experiment, healthy sea cucumbers were randomly divided into three groups (siAjNotch-1 group, siAjNotch-2 group, and siNC group). The sea cucumbers with excised ventral papillae from each group were evenly distributed into five groups, and each sea cucumber was injected with the corresponding transfection solution every 2 days over a 28-day papilla regeneration period [44]. The initial injection was performed at 0 dpe, and the final injection was administered at 26 dpe. Throughout the papilla regeneration process, a completely dark environment was maintained, with daily fresh seawater exchange and regular feeding. Papilla samples were collected from sea cucumbers in each group at 0, 7, 14, 21, and 28 dpe, and subsequently stored at −80 °C for subsequent gene expression analysis. After determining the interference efficiency, siAjNotch-2 was selected as the targeted disruptor for the *AjNotch* in the subsequent experimental phase.

To further clarify the regulatory effect of the Notch signaling pathway on sea cucumber papilla regeneration under LED light, a 28-day sea cucumber papilla regeneration experiment was conducted. Prior to the experiment, four groups were established: a completely dark-Control group (0 lx, Control-siNC group), a completely dark-RNAi group (0 lx, Control-siAjNotch group), a LED light-Control group (2000 lx, LED-siNC group), and a LED light-RNAi group (2000 lx, LED-siAjNotch group). A total of 72 healthy *A. japonicus* were evenly assigned to four experimental groups, with three independent biological replicates per group and six individuals per replicate. The ventral papillae of all individuals were uniformly excised before the experiment. The siRNA mixture and injection procedure were identical to those used in the preceding interference experiment. interference experiment. During the gene silencing experiment, the photoperiod, water exchange frequency, and feeding regime were consistent with those described in Section 4.3. On the 28th day of the gene silencing experiment, regenerating papilla tissues were collected separately from each experimental group during the light phase and dark phase for subsequent gene expression detection and EdU staining observation. Specifically, 6 sea cucumbers were sampled from each independent replicate, with 3 individuals sampled from the light phase and 3 individuals sampled from the dark phase.

### 4.6. Histomorphological and Cell Proliferation Analysis

Upon completion of the experiment, regenerating papilla samples were randomly selected from 9 sea cucumbers across the three parallel groups (3 individuals per group) for subsequent histological staining assays and analyses. Samples were fixed in 4% paraformaldehyde dissolved in 1× PBS (pH 7.2) at 4 °C for 24 h, then rinsed three times with 1× PBS, 5 min per rinse. Subsequently, samples were subjected to gradient dehydration (75% → 95% → absolute ethanol, 5 min per step), xylene clearing (15 min per step), and paraffin infiltration at 60 °C (30 min per step). After paraffin embedding, 5 μm-thick longitudinal tissue sections were prepared and baked at 60 °C for 2 h.

Hematoxylin–Eosin (HE) Staining: Sections were dewaxed and rehydrated using an environment-friendly dewaxing solution (Servicebio, Cat. No. G1128), treated with high-definition staining pretreatment solution (from HE staining kit, Servicebio, Cat. No. G1076) for 1 min, stained with hematoxylin for 3 min, differentiated with differentiation solution (Servicebio, Cat. No. G1039) for 3–5 s, rinsed with running water for bluing, stained with eosin for 15 s, dehydrated, cleared, and mounted with neutral gum.

Masson’s Trichrome Staining: After dewaxing and rehydration, sections were incubated with Solution A from the Masson staining kit (Servicebio, Cat. No. G1006) at 65 °C for 30 min, stained with a mixture of Solutions B and C for 1 min, incubated with Solution D for 6 min, stained with Solution F for 20 s, rinsed with 1% glacial acetic acid, dehydrated, cleared, and mounted with neutral gum.

5-ethynyl-2′-deoxyuridine (EdU) Staining: EdU staining was performed to detect cell proliferation during papilla regeneration. EdU was injected intraperitoneally 24 h prior to sampling at each papilla regeneration time point. Staining protocol: After dewaxing, sections were permeabilized with permeabilization buffer (Servicebio, Cat. No. G1204) for 20 min and rinsed three times with 1 × PBS. Subsequently, 60 μL of Click-iT EdU-488 staining reaction solution (Servicebio, Cat. No. G1601) was added to each section, followed by incubation in the dark for 30 min. Sections were then stained with DAPI (Servicebio, Cat. No. G1012) for 10 min, rinsed with 1× PBS, mounted with anti-fluorescence quenching mounting medium (Servicebio, Cat. No. G1401), and observed under a fluorescence microscope (Nikon, Tokyo, Japan). For quantitative analysis, five non-overlapping visual fields were randomly selected from each section in a blinded manner to avoid bias. A fixed total of 300 nuclei were counted in each field, and the mean value of the five fields was used for statistical analysis.

### 4.7. Data Analysis

All experimental data were expressed as mean ± S.D. OriginPro 2021 (OriginLab, Northampton, MA, USA) was used for chart preparation. We first confirmed that our data were normally distributed and homogeneous using the Shapiro–Wilk test and Levene’s test. Tukey and one-way ANOVA were employed for statistical analysis in SPSS 22.0 (IBM, Armonk, NY, USA), with significant differences at *p* < 0.05 and extremely significant differences at *p* < 0.01.

## 5. Conclusions

This study explored the functional roles of the Notch signaling pathway in *A. japonicus* papilla regeneration. We first detected the expression patterns of Notch signaling pathway-related genes (*AjNotch*, *AjSu(H)*, and *AjHes1*) during papilla regeneration. Results demonstrated that Notch signaling is involved in the papilla regeneration process of *A. japonicus* and positively modulates the regenerative development of its papillae. LED light experiments identified an optimal light intensity for promoting papilla regeneration, while *AjNotch* interference inhibited papilla regeneration with concurrent downregulation of the Notch signaling pathway and its downstream proliferation-related genes. In summary, the present study advances our understanding of the functional roles of *AjNotch* during papilla regeneration in the sea cucumber *A. japonicus*, clarifies the critical involvement of the Notch signaling pathway in papilla regeneration in echinoderms, and identifies 2000 lx LED light as a controllable artificial intervention strategy to enhance papilla regeneration in *A. japonicus*.

## Figures and Tables

**Table 1:** The qRT-PCR primers were as follows.

Primers	Sequence (5′-3′)
*AjNotch*-F	AAGGGGTGGACTGTGAGAGAGAA
*AjNotch*-R	GCAAGTGAAGGAGCCCAATGAAT
*AjSu(H)*-F	CGTTCACGGAGGTCTCTTAGAT
*AjSu(H)*-R	CGGAGTTTGGCGGAGTATTTGG
*AjHes1*-F	GCCGTTGGATAAACCCAGACCC
*AjHes1*-R	GACGTGACGCCCGTGAACACTC
*AjCyclinA*-F	TATCAAGGCCAGCGACGAAGGAG
*AjCyclinA*-R	GGAGATGCAATGTGTGTCGAGCC
*AjCyclinD*-F	GAAACGGACTCAGTACCCC
*AjCyclinD*-R	AAACATCCACTCGACCACC
*AjCDK8*-F	CTTGAAGCAAATAGAGGG
*AjCDK8*-R	ATTGTGATTGGAACGGAG
*Cytb*-F	TGAGCCGCAACAGTAATC
*Cytb*-R	AAGGGAAAAGGAAGTGAAAG

**Table 2:** RNA oligonucleotides used in the study.

Primers	Sequence (5′-3′)	Appilication
siNC-F	UUCUCCGAACGUGUCACGUTT	RNAi
siNC-R	ACGUGACACGUUCGGAGAATT	
siAjNotch-1-F	CGAAGGUGAUUACUGUCAATT	RNAi
siAjNotch-1-R	UUGACAGUAAUCACCUUCGTT	
siAjNotch-2-F	GCGUGUGUGCAUUAGGUUATT	RNAi
siAjNotch-2-R	UAACCUAAUGCACACACGCTT	

## Data Availability

The original contributions presented in the study are included in the article; further inquiries can be directed to the corresponding authors.

## Supplementary Materials

The following supporting information can be downloaded at: https://www.mdpi.com/article/10.3390/ijms27094105/s1.
